# Embryo movement is required for limb tendon maturation

**DOI:** 10.3389/fcell.2024.1466872

**Published:** 2024-11-05

**Authors:** Rebecca A. Rolfe, Ebru Talak Bastürkmen, Lauren Sliney, Grace Hayden, Nicholas Dunne, Niamh Buckley, Helen McCarthy, Spencer E. Szczesny, Paula Murphy

**Affiliations:** ^1^ Zoology, School of Natural Sciences, Trinity College Dublin, University of Dublin, Dublin, Ireland; ^2^ School of Mechanical and Manufacturing Engineering, Dublin College University, Dublin, Ireland; ^3^ Advanced Materials and Bioengineering Research Centre (AMBER), Trinity College Dublin, University of Dublin, Dublin, Ireland; ^4^ Advanced Manufacturing Research Centre (I-Form), School of Mechanical and Manufacturing Engineering, Dublin City University, Dublin, Ireland; ^5^ School of Pharmacy, Queens University Belfast, Belfast, United Kingdom; ^6^ Department of Biomedical Engineering, Pennsylvania State University, University Park, PA, United States; ^7^ Department of Orthopaedics and Rehabilitation, Pennsylvania State University, Hershey, PA, United States

**Keywords:** tendon maturation, collagen fiber alignment, muscle paralysis, yes-associated protein, skeletal development, embryonic movement, cellular organization

## Abstract

**Introduction:**

Following early cell specification and tenocyte differentiation at the sites of future tendons, very little is known about how tendon maturation into robust load-bearing tissue is regulated. Between embryonic day (E)16 and E18 in the chick, there is a rapid change in mechanical properties which is dependent on normal embryo movement. However, the tissue, cellular and molecular changes that contribute to this transition are not well defined.

**Methods:**

Here we profiled aspects of late tendon development (collagen fibre alignment, cell organisation and Yap pathway activity), describing changes that coincide with tissue maturation. We compared effects of rigid (constant static loading) and flaccid (no loading) immobilisation to gain insight into developmental steps influenced by mechanical cues.

**Results:**

We show that YAP signalling is active and responsive to movement in late tendon. Collagen fibre alignment increased over time and under static loading. Cells organise into end-to-end stacked columns with increased distance between adjacent columns, where collagen fibres are deposited; this organisation was lost following both types of immobilisation.

**Discussion:**

We conclude that specific aspects of tendon maturation require controlled levels of dynamic muscle-generated stimulation. Such a developmental approach to understanding how tendons are constructed will inform future work to engineer improved tensile load-bearing tissues.

## 1 Introduction

Tendons and ligaments are connective tissues within the musculoskeletal system that must sustain large mechanical loads. While ligaments connect bone to bone, tendons play a key role in integrating the musculoskeletal system, transmitting the loads produced by muscle contraction to the skeleton to facilitate movement. Despite their honed hierarchical structures that provide the robust tensile strength required, tendons and ligaments are prone to injury and wear and tear, leading to pain and disability (Millar et al., 2021). This presents acute clinical challenges due to the limited healing capabilities of such hypocellular and hypovascular tissues, with long-term morbidity at the site even when surgical repair is attempted. For all these reasons, tendons are prime targets for tissue engineering approaches; yet, to date, attempts have failed to develop structurally robust tissue with appropriate biomechanical properties for regenerative therapies. A developmental biology approach is required to understand each of the steps involved and the mechanisms that drive the natural assembly of these highly ordered and structurally important tissues in order to recapitulate or mimic the processes *in vitro* (Glass et al., 2014; Szczesny and Corr, 2023).

Although much has been uncovered about the origin of progenitor cells and early tendon development, less is known about important steps during later development when rapid changes occur in macroscale mechanics to form a tissue capable of bearing a load (McBride et al., 1988). Tendon primordia are already detected in place in the early chick limb bud (Kardon, 1998; Roddy et al., 2011), and key signaling pathways and transcription factors required for early development have been identified, such as scleraxis (Scx) and Egr1, which responds to TGFβ, FGF signaling, and mechanical cues (Gaut et al., 2016; Havis et al., 2016; Schweitzer et al., 2001). The transmembrane glycoprotein tenomodulin (Tnmd) is a regulator of cell proliferation and collagen fibril maturation (Docheva et al., 2005; Shukunami et al., 2006), while Mohawk (Mkx) is a transcription factor that also regulates tendon differentiation (Liu et al., 2015). The predominant collagen expressed is collagen 1, with minor contributions from collagens 3 and 5 (Silver et al., 2003). During development, tenocytes align longitudinally, forming linear channels for collagen deposition and fibril formation (Bobzin et al., 2021; Kalson et al., 2010; Richardson et al., 2007). Collagen fibrils align into fibers, which are bundled into fascicles, forming the tendon. It is this hierarchical structure of the extracellular matrix (ECM) that gives the tendon its biomechanical properties. The ratio of the cell to extracellular matrix changes as tendons mature, and at a critical timepoint (embryonic day (E) 16–18 in the chick), there is a rapid increase in fiber stiffness and strength (Birk et al., 1995; McBride et al., 1988; Peterson et al., 2021). Very little is known about the cellular and molecular changes that take place and what drives these rapid changes in biomechanical properties although the transition is dependent on embryo movement (Peterson et al., 2021; Peterson et al., 2024).

Embryo movement is essential for the normal development of multiple components of the musculoskeletal system, including bone size, shape and mechanics, and joint formation (Felsenthal and Zelzer, 2017; Murphy and Rolfe, 2023). While mechanical forces from movement are important at multiple points in tendon development (Edom-Vovard et al., 2002; Germiller et al., 1998), designing the experiments to specifically examine the critical period of transition in biomechanical properties has shown that embryonic immobilization results in a reduced mechanical modulus and failed developmental maturation (Pan et al., 2018; Peterson et al., 2021; Peterson et al., 2024). We have previously shown that rigid paralysis leads to a reduction in the size of tendons, fiber diameters, and fibril lengths (Peterson et al., 2021; Peterson et al., 2024). Further investigation of the impact of immobilization is needed to shed light on the cellular, molecular, and biomechanical changes that are critical for tendon maturation.

Very little is known about the molecular mechanisms that interpret mechanical cues generated by movement in the skeletal system. We have previously implicated Wnt and BMP signaling as sensors that respond in the forming joint region (Rolfe et al., 2018; Singh et al., 2018) and yes-associated protein (YAP) signaling in localized growth required for bone shape (Shea et al., 2020). YAP is of particular interest as it is known to respond to external environmental cues in a number of contexts (reviewed by Panciera et al., 2017), including cell fate decisions in the early embryo (reviewed by Yildirim et al., 2021) and in the control of tissue structure and size in developing the lung, kidney, brain epidermis, liver, bone, teeth, and heart (reviewed by Davis and Tapon, 2019; Mia and Singh, 2019; Pibiri and Simbula, 2022; Yang et al., 2023; Zhao et al., 2021). The famous work of Piccolo and colleagues demonstrated that YAP is regulated by the mechanical environment; mesenchymal stem cells grown on rigid surfaces showed increased nuclear YAP activity and osteogenic differentiation, while softer matrices resulted in cytoplasmic YAP and adipogenesis (Dupont et al., 2011). Very little is known about YAP signaling during tendon development, apart from transcriptomic evidence of YAP target gene expression during late stages (Yeung et al., 2015); however, there is evidence that YAP can influence tenogenesis *in vitro*, with inhibition of YAP downregulating tendon-related gene expression in differentiating mesenchymal stem cells (Li et al., 2022), enhanced nuclear translocation of YAP reported to promote tenogenic differentiation (Tao et al., 2021; Tomás et al., 2019), upregulation of YAP following treatment with a novel exercise-induced myokine (irisin) that promotes tenogenic proliferation and differentiation in culture (Xu et al., 2022), and improved performance of progenitor cells following YAP activation in culture (Lu et al., 2023). Furthermore, YAP is critical to sensing tensional homeostasis in mature tendons (Jones et al., 2023). YAP is, therefore, a prime candidate for investigation as a mediator of mechanosensitive tendon maturation.

To enhance understanding of the tissue, cellular, and molecular level changes that take place during tendon maturation, in this study, we examine embryonic chick metatarsal tendons across late stages of development, quantifying changes in collagen fiber alignment, cell density, nuclear shape, and YAP pathway component levels. We expand our previous analysis (Peterson et al., 2021; Peterson et al., 2024) of the impact of immobilization on tendon maturation by comparing rigid and flaccid paralysis across multiple tendons. Rigid paralysis is induced by treatment with decamethonium bromide (DMB), a neuromuscular blocking agent that eliminates dynamic stimulation while maintaining constant static tension, whereas flaccid paralysis, induced by pancuronium bromide (PB), results in the loss of both static and dynamic forces (Osborne et al., 2002; Rolfe et al., 2017). We show the effects of immobilization on tendon size, spacing, collagen fiber alignment, and cellular organization. Having previously shown that YAP signaling is altered in the skeletal rudiments of muscle-less mouse embryos (Shea et al., 2020), we explored, for the first time, the potential involvement of YAP signaling in the mechanoregulation of embryonic tendon maturation.

## 2 Materials and methods

### 2.1 Egg incubation and *in ovo* movement manipulation

Fertilized eggs (Ross 308, supplied by Allenwood Broiler Breeders) were incubated at 37.7°C in a humidified incubator. Work on chick embryos does not require a license from the Irish Ministry of Health under European Legislation (Directive 2010/63/EU); all work on chick embryos was approved by the Trinity Ethics Committee. Following 3 days of incubation, 5 ml of albumen was removed from each egg using an 18-gauge needle. Embryos were euthanized daily from E14 (HH40) to E20 (HH45) to assess the structural and molecular changes that occur during late tendon development.

Immobilization treatments consisted of daily application of 0.2% decamethonium bromide (rigid paralysis) or 0.2% pancuronium bromide (flaccid paralysis) (both Sigma-Aldrich) in sterile Hank’s balanced salt solution (HBSS) (Gibco) plus 1% of antibiotic/antimycotic (aa) (penicillin, streptomycin, and amphotericin B; Sigma-Aldrich), dripped directly onto the vasculature of the chorioallantoic membrane through the “windowed” egg. Immobilization began at E16 (100 µL of 0.2% DMB or 0.2% PB) with subsequent daily treatments (50 µL) until harvest. Controls were treated with equal volumes of HBSS plus aa. The experiment was repeated independently three times. Control and immobilized embryos were sacrificed at E20 (HH45) to investigate the structural and molecular effects of immobilization.

Each egg was placed on ice for at least 15 min prior to euthanasia via decapitation. Embryos were dissected in ice-cold phosphate-buffered saline (PBS). Each embryo was staged using the Hamburger and Hamilton criteria (Hamburger and Hamilton, 1992). Limbs were dissected and either fixed in 4% paraformaldehyde (PFA) in PBS at 4°C at least overnight, or metatarsal ankle tendons were sub-dissected and placed in TRIzol for subsequent protein and RNA extraction.

### 2.2 Histological tissue processing

Fixed hindlimbs were placed in 10% EDTA (pH 7.4) for at least 7 days at 4°C for decalcification and subsequently dehydrated through a graded series of ethanol (EtOH) (25%, 50%, and 70%) for a minimum of 1 × 1 h washes. Limbs were stored in 70% EtOH prior to processing for paraffin embedding or cryosectioning. One hindlimb from each specimen was processed for paraffin sectioning. Paraffin processing involved subsequent 2 × 1 h washes in 100% EtOH, 2 x Histo-Clear (National Diagnostics) washes (1 h each), and a minimum of 5 × 1 h paraffin washes prior to embedding in paraffin. A full series of longitudinal (8 µm) sections through the ankle joint and a series of cross sections (8 µm) through the midpoint of the tarsometatarsal region at a distance of approximately 1–2 mm proximal to digit two of the foot were prepared for each specimen. Paraffin sections were dewaxed, rehydrated, and stained to highlight connective tissue using Masson’s trichrome (Sigma-Aldrich, HT15) and further processed for protein localization (cf 2.3 and 2.4).

Hindlimbs stored in 70% EtOH processed for cryosectioning were rehydrated and cryoprotected in 30% sucrose in PBS overnight. Longitudinal sections (10 µm) were prepared for immunolabeling.

### 2.3 Picrosirius red staining and polarized light microscopy

**FIGURE 1:**
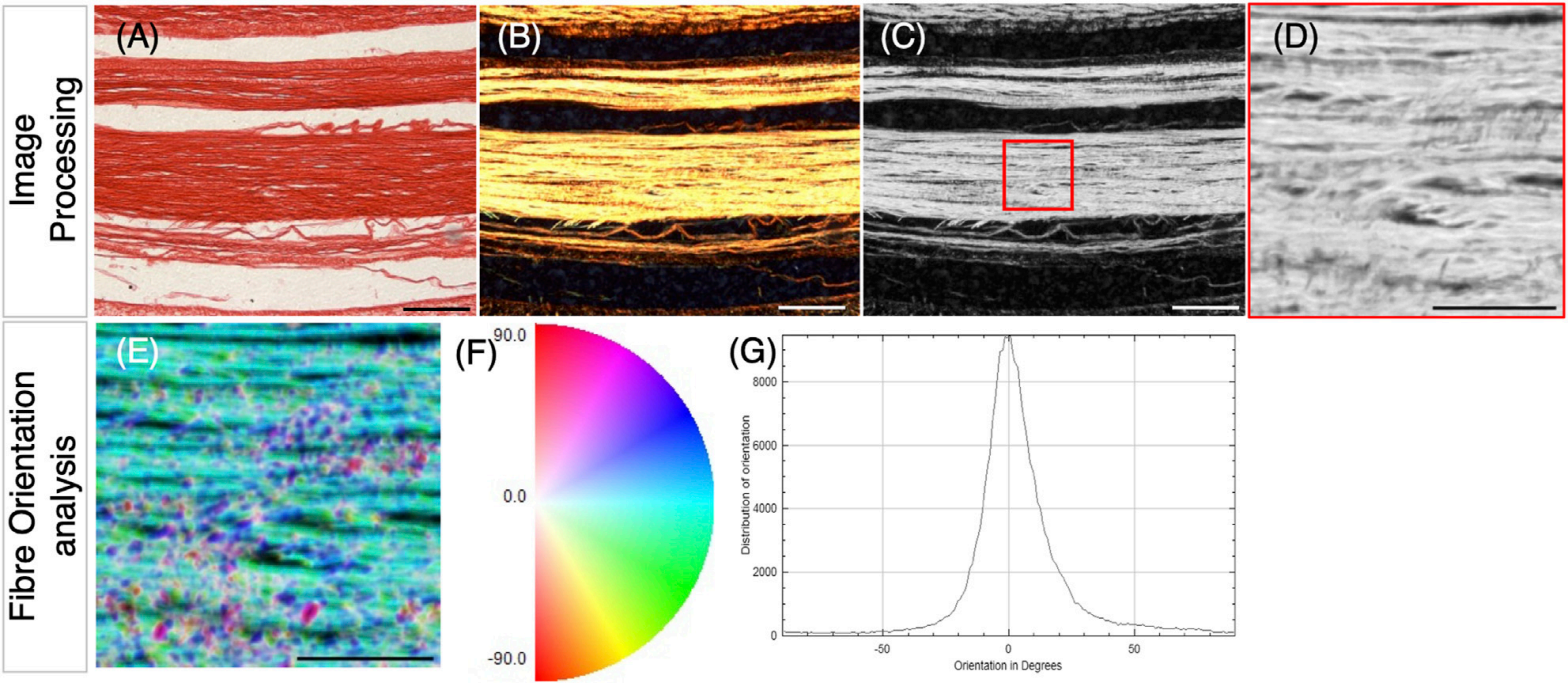
Methodology for collagen fiber orientation analysis, following picrosirius red staining of tendon sections and polarized light microscopy. **(A)** Example brightfield image of a longitudinal section through the metatarsal tendons (E18) stained with picrosirius red. **(B–D)** Polarized light images of the tendon shown in **(A)**. **(B)** Merging of two polarized light images; the second was taken following the rotation of the analyzer filter by 45° (to capture more of the birefringence). **(C)** 8-bit grayscale conversion of **(B),** showing the selection of the 500 × 500 pixels (4.4 pixels/µm) with ROI for analysis, placed within a fiber bundle (red square). **(D)** Selected ROI. **(E–G)** Analysis of fiber orientation within the ROI shown in **(D)** using the OrientationJ plugin with ImageJ. This generates a fiber orientation color map **(E)**, where blue/cyan indicates fibers oriented close to 0⁰ and pink/red indicates fiber oriented ∼90⁰ **(F)**. **(G)** Using the color map information, a histogram of local angles can be generated, representing the mean and standard deviation of orientations for that ROI. Data were extracted from the generated tables to represent the proportion of fibers in orientation groupings (within ± 10°; ± 10–20°; and ± 20–30°), as shown in box plots in Figures 2, 4. Scale bars **(A–C),** 100 µm and **(D–E)**, 50 µm.

Collagen fiber alignment was examined in longitudinal sections of metatarsal tendons across development (E14, E16, E18, and E20) and after immobilization. Collagen was stained using Picrosirius red (0.1% of Direct Red 80 in saturated picric acid solution (Sigma P6744)) for 1 h, and the samples were examined under polarized light (Olympus BX41TF Microscope fitted using polarizer and analyzer filters). Images were captured using Ocular software. The sections were consistently oriented with the tendon’s long axis running left to right on the imaging screen, and images were captured with a ×20 objective, first under white light to image the ROI, followed by two polarized light images (Figure 1A). The polarized light images were selected by first setting the polarizer filter to 0° and rotating the analyzer filter until the brightest birefringence pattern was visible (image 1; angle 0°) and then moving the polarizer filter to 45° (image 2; angle 45°). Using ImageJ, the two images were merged and converted to 8-bit, and the central portion of the tendon was cropped to 500 × 500 pixels (4.4 pixels/µm) for further analysis (Figures 1B–D). Fiber alignment analysis was performed on each ROI using the Plugin OrientationJ tool in ImageJ (Figures 1E–G) (Rezakhaniha et al., 2012), specifically using “distribution for histogram” and “analysis for color map” functions. This produces a color map of orientations and allows a histogram of local orientations to be generated (Figure 1G). A minimum of three ROIs were measured for each independent biological sample (chick embryo) across developmental times and after treatment. Specifically, for E14 and E20, 10 ROIs were analyzed across each of 4 embryos; for E16, 10 ROIs across each of 3 embryos; and for E18, 17 ROIs across each of 5 embryos. For the comparison of control and immobilized, 49 ROIs across each of 14 control embryos, 27 ROIs across 7 rigid immobilized embryos, and 23 ROIs across 6 flaccid immobilized embryos were analyzed. Fiber alignment analysis data were represented as the proportion of fibers within three orientation categories (±10°, ±10–20°, and ±20–30°) across each group for comparison and statistical analysis. Statistical analysis was carried out using univariate ANOVA, followed by Tukey’s *post hoc* tests (*p ≤ 0.05, **p ≤ 0.01, and ***p ≤ 0.001).

### 2.4 Immunohistochemistry and collagen detection

Heat-mediated antigen retrieval was performed on histological sections to improve detection for all the following target proteins using 0.01 M sodium citrate (pH 8) for 20 min at 90°C. Collagen was detected using a fluorescently labeled CNA35 collagen binding protein (CNA35-eGFP) (plasmid DNA kindly provided by Maarten Merkx). The recombinant protein was expressed, purified from *E. coli* (1–2 mg/mL), and utilized as previously described (Mohammadkhah et al., 2017). Blocking was performed using 1% bovine serum albumin (BSA) in PBS for 1 h at RT, and incubation in binding protein was carried out at a dilution of 1:20–1:50 in the blocking solution overnight at 4°C. Following subsequent PBS washes, mounting with DAPI nuclear stain was performed (ProLong Gold Antifade Reagent with DAPI, Life Technologies). Following microscopic imaging (detailed below), fluorescent intensity analysis was carried out with ImageJ using ×20 magnification with a minimum of five ROIs per image and five technical replicates per biological sample; the mean gray level represents fluorescence intensity.

For YAP and pYAP tissue-level detection, following antigen retrieval, paraffin sections were processed, undergoing heat-mediated antigen retrieval, blocked in 5% goat serum in TBS with 0.1% Tween (TBST) and primary antibody, incubated overnight at 4°C. The following primary antibodies were used: mouse monoclonal anti-YAP ((63.7) Santa Cruz- sc101199; 1:200 blocking solution) and rabbit polyclonal anti-phospo-YAP, Ser127 (Cell Signaling, cat. No 4911; 1:200 blocking solution). Colorimetric secondary detection was performed for whole tissue-level analysis using goat anti-mouse (Cell Signaling- 7056S; 1:2000 blocking solution) or anti-rabbit IgG-alkaline phosphatase (Invitrogen- G21079; 1:200 blocking solution) with sections equilibrated to pH 9.5 in NMT buffer (0.1 M Tris at pH 9.5, 0.05M MgCl_2_, 0.1% Tween, or 0.1 M NaCl), then developed using NBT (nitro blue tetrazolium)/BCIP (5-bromo-4-chloro-3-indoly phosphate) and diluted in NMT. Colorimetric samples were mounted using Aqua-Poly/Mount (Polysciences).

For GAPDH detection, paraffin sections were blocked in 5% bovine serum albumin in PBS, incubated with primary antibodies, and mouse monoclonal anti-GAPDH (Mouse, Novus Bio, NB300-221, 1:200) overnight at 4°C. Fluorescent secondary detection was performed using Alexa Fluor 488 goat anti-mouse IgG (Invitrogen, A11001, and 1:250 blocking solution) and mounted using ProLong Gold Antifade Reagent with DAPI (Life Technologies).

Tissue sections were photographed under brightfield and fluorescent microscopy (as appropriate) using the Olympus DP72 Camera and cellSens software (v1.6).

### 2.5 Image analysis and quantification of morphological and cellular parameters

The cross-sectional areas of the Achilles tendon, flexor digitorum longus (FDL), and the flexor digitorum brevis (FDB) digit II tendons were quantified from embryos at E20 (HH45), following rigid and flaccid immobilization and experimental controls (n = 7–12 control, n = 8–11 rigid, and n = 7–8 flaccid-immobilized biological replicates from 2–4 independent experiments) using measurements from 7–20 adjacent Masson’s trichrome stained cross-sections through a 560–1,200 µm portion of the medial tarsometatarsal region of interest (ROI) for each replicate embryo. The boundary of collagen-positive (blue) staining defined the boundary of the tendon for measurements (as shown in Figure 3A). The cross-sectional area of the Achilles tendon, FDL2 tendon, and FDB2 tendon were quantified independently using ImageJ (v2.1.0/1.53c). The width of the Achilles tendon was also measured (Supplementary Figure S1). The same medial ROI was used to assess the spacing between the FDL and FDB tendons following immobilization. For each section of every sample, 3–8 measurements were taken across the extent of the interface (where approximately parallel) between the tendons and averaged to represent the spacing between tendons. Spacing measurements represent n = 7 control, n = 8 rigid, and n = 7 flaccid biological replicates. The effects of immobilization were also analyzed in the knee region, specifically on the patellar tendon (Supplementary Figure S1).

Nuclear imaging and analysis were performed using ×100 magnification images of DAPI-stained tendons across normal development E14–E20 and following immobilization using ImageJ. Cell densities were quantified using the cell counter function in a field of view equal to 137.412 µm^2^ (Figure 4C) across 3–5 technical replicates per developmental stage (embryos analyzed per stage: E14 n = 4; E16, n = 3; E18, n = 4; and E20, n = 3) or treatment group (embryos analyzed per group; control, n = 12; rigid, n = 7; and flaccid, n = 6). The analysis of nuclear shape was performed using original files (Supplementary Figure S3A) cropped to define an ROI of 5 µm × 4 µm (Supplementary Figure S3B). Cropped images were split into component color ‘channels,’ and the blue channel (Supplementary Figure S3C) was determined to have the best signal-to-noise ratio and so was used for further analysis. Images were converted to 8-bit by setting a minimum threshold using the auto threshold function as a guideline. This converted every pixel above the threshold in the grayscale blue channel to black and every pixel below the threshold to white (Supplementary Figure S3D). The appropriate threshold values were determined independently for each biological replicate due to the unavoidable variability in staining contrast or intensity and image capture. Any background noise or out-of-focus nuclei were removed either manually or using the despeckle function, and converged nuclei were separated (Supplementary Figure S3E). Nuclei were measured using the analyze particle function (Supplementary Figure S3F), with a minimum particle size of 0.05 μm^2^. Nuclei touching the edge of the ROI were excluded to prevent inaccurate shape measurements. The analyze particle function generated the shape measures of circularity and aspect ratio. Circularity was calculated as 4π times the area divided by the perimeter squared. A perfect circle has a circularity value of 1.0; as the shape becomes more ellipsoid, its circularity value decreases (Supplementary Figure S3G).

The transverse distances between cell nuclei in adjacent aligned columns in the longitudinal tissue plane were calculated from 5 µm × 4 µm cropped images for each developmental stage and treatment group. A minimum of three images per biological replicate were used to assess the distance between nuclei. Measurements were made between the edges of adjacent nuclei using the line and measure function in ImageJ, and each individual measurement was treated independently. The total number of measurements across independent biological replicates is as follows: E14 = 110 from 4 biological replicates, E16 = 95 from 3 replicates, E18 = 106 from 3 replicates, E20 = 87 from 3 replicates, control = 260 from 12 replicates, rigid immobilized = 156 from 7 replicates, and flaccid immobilized = 104 from 6 replicates.

### 2.6 Protein and mRNA quantification from dissected tendons

Tendons ventral to the ankle joint, including the flexor halluces brevis, the flexor perforans et perforatus, and the flexor digitorum, were sub-dissected from hindlimbs between the ankle joint and proximal to the footplate. Tendons were pooled from each embryo, placed in TRIzol (Thermo Fisher Scientific), and stored at −70°C. Mechanical homogenization was performed using polypropylene pestles and high-speed vortexing. Protein extraction was performed according to the manufacturer’s protocol (TRIzol; Thermo Fisher Scientific), resuspended in 8 M urea with 1% SDS in a 0.05 M of Tris (pH = 8) solution, quantified (Qubit 2.0 fluorometer, Invitrogen), and stored at −20°C. For western blotting, 40 µg of protein were separated on a 10% pre-cast polyacrylamide gel (Bio-Rad) and transferred to PVDF membranes. PVDF membranes (0.45 µm, Bio-Rad, Trans-Blot Turbo PVDF (Cat#10026934)) were blocked using 5% milk powder in TBST and incubated in 1:1,000 anti-YAP ((63.7) Santa Cruz-sc101199), 1:1,000 anti-phospo-YAP (Cell Signaling, cat. No 4911), or 1:1,000 anti GAPDH (NB300-221 Novus Bio). Detection and visualization were performed using fluorescent detection with Li-COR antibodies (1:10,000 goat-anti-mouse IRDye 680RD and goat-anti-rabbit IRDye 800CW) and a Li-COR Odyssey system (Odyssey XF). Densitometry analysis was performed using ImageJ software for YAP and pYAP protein levels normalized to GAPDH protein levels, comparing developmental timepoints (E14–E20) and between control and immobilized samples at E20 (n = 12 (control), n = 11 (rigid), and n = 3 (flaccid) independent embryos in each case).

RNA was extracted from the stored TRIzol tissue homogenate using chloroform and purified using a TRIzol PureLink Kit (Invitrogen), following the manufacturer’s instructions. Typically, mRNA was re-suspended in 30 µL of nuclease-free water and quantified using the Qubit 2.0 Quantitation System (Invitrogen). mRNA was reverse-transcribed using a standard quantity of total RNA (500 ng) and High-Capacity cDNA Reverse Transcription Kit (Applied Biosystems™: 4368814) and diluted at a ratio of 1:5 with RNase-free water. Chick primers for genes of interest (Table 1) were synthesized by Merck. *GAPDH* was used as the normalizer transcript. Real-time PCR quantification was performed using an ABI 7500 Sequence Detection System (Applied Biosystems) with SYBR Green gene expression quantification (Applied Biosystems). A measure of 5 μL of cDNA preparation was diluted at a ratio of 1:5 with RNase-free water, followed by 10 μL of 2x SYBR Green PCR Master Mix (Applied Biosystems), 0.5 µL (10 μM) of each primer, and 4 µL RNase-free water. Samples were assayed in triplicate in one run (40 cycles), which consists of three stages: 95°C for 10 min, 95°C for 15 s for each cycle (denaturation), and 60°C for 1 min (annealing and extension). Data were analyzed using relative quantification and the Ct method, as described previously (Livak and Schmittgen, 2001). The level of gene expression was calculated by subtracting the averaged Ct values (Ct is the threshold cycle) for *GAPDH* from those of the genes of interest. The relative expression was calculated as the difference (ΔΔCt) between the Ct of the test stage or condition (immobilized) and that of the compared stage or the control condition. The relative expression of genes of interest was calculated and expressed as 2^−ΔΔCT^. Relative quantification values are presented as fold changes plus/minus the standard error of the mean relative to the comparator group, which was normalized to 1.

**TABLE 1:** Details of primers used in qRT-PCR.

Gene name	Primer sequence	Reference sequence	Amplicon size
GAPDH	Fwd: 5′GGC​TGA​GAA​CGG​GAA​ACT​TG 3′	NM_204305.2	51bp
	Rv: 5′TGG​AAG​ATA​GTG​ATG​GCG​TGC 3′		
Scx	Fwd: 5′CAC​CAA​CAG​CGT​CAA​CAC​C 3′	NM_204253.1	88bp
	Rv: 5′CGT​CTC​GAT​CTT​GGA​CAG​C 3′		
Tnmd	Fwd: 5′ CTG​CCG​CTT​TTT​GGA​TGC​AG 3′	NM_206985.3	120bp
	Rv: 5′GAC​ACC​AAA​GAG​CAC​GAG​GA 3′		
Mkx	Fwd: 5′GAA​CAC​AGT​CAG​GCA​ACC​AGA​CC 3′	XM_015282064.4	120bp
	Rv: 5′CCA​TCT​TCA​GAG​CAC​GAG​TCA​TCA​C 3′		
Col1a1	Fwd: 5′TGC​TGT​TGA​TAG​CAG​CGA​CT 3′	NM_001396622.1	184bp
	Rv: 5′GTG​TCC​TCG​CAG​ATC​ACC​TC 3′		
Col3a1	Fwd: 5′ TTC​AGG​AGC​AAG​GGG​TCC​ACC 3′	NM_205380.3	198bp
	Rv: 5′AGG​GAA​GCT​ACG​CCA​CCA​CCA 3′		
Col5a1	Fwd: 5′CGA​CCT​GGA​TAA​AGA​CTT​CAC​CG 3′	NM_204790.4	140bp
	Rv: 5′GGC​CTC​CGA​TCC​CTT​CGT​A 3′		
Ctgf	Fwd: 5′AAG​ACA​CTT​ACG​GCC​CAG​AC 3′	NM_204274.2	59bp
	Rv: 5′AGT​AGT​CTG​CAC​CAG​GCA​AT 3′		
Cyr61	Fwd: 5′GGA​ACA​ACG​AGC​TGA​TTG​CC 3′	NM_001031563.2	67bp
	Rv: 5′CGG​CTC​GGA​TCC​AAA​AAC​AG 3′		
Ankrd1	Fwd: 5′GAA​ATG​CAG​GAA​AGT​GAA​AGC 3′	NM_204405.2	94bp
	Rv: 5′TGC​AGC​TCT​GAA​GAA​CAT​GG 3′		

### 2.7 Statistical analysis

A range of 3–6 independent biological replicates (embryos) were analyzed for all normal development stages and between 8–15 independent biological replicates for control and immobilized specimens across 2–4 independent experiments. Specifically for morphological analysis, 12 controls and 11 rigidly and 8 flaccidly immobilized biological replicates were assessed. For PLM, 14 controls and 7 rigidly and 6 flaccidly immobilized biological replicates were measured. For nuclear comparisons, 12 controls and 7 rigidly and 6 flaccidly immobilized biological replicates were assessed, with 604 nuclei from control, 356 nuclei from rigid, and 308 nuclei from flaccid immobilized replicates analyzed for nuclear shape. For all data, SPSS statistics (IBM^®^, v.27) was used for statistical analysis. Significance was determined using one-way ANOVA, followed by Tukey’s *post hoc* test with a 95% confidence interval. *p*-values ≤0.05 were considered significant.

## 3 Results

### 3.1 Collagen fiber alignment increases during tendon maturation as tenocytes stack into parallel columns

**FIGURE 2:**
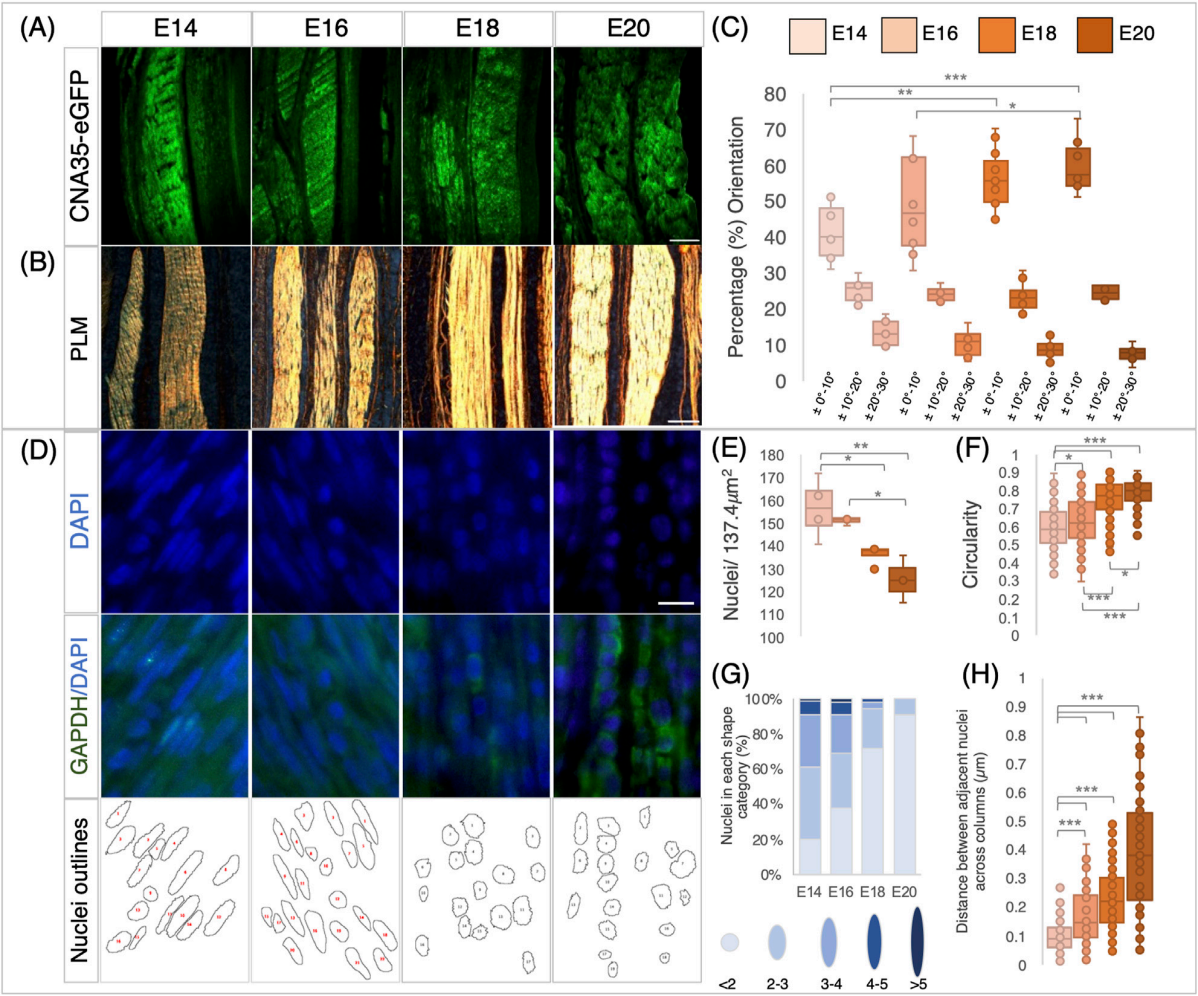
Changes in collagen deposition and cellular organization in chick metatarsal tendons across late stages of development. **(A)** Visualization of collagen deposition in longitudinal sections through embryonic chick metatarsal tendons using a fluorescently tagged pan collagen-binding protein (CNA35-eGFP) at indicated developmental timepoints. **(B)** Collagen fiber bundles visualized by picrosirius red staining and PLM in longitudinal sections of metatarsal tendons. **(C)** Collagen fibers become more aligned across development. Quantification of fiber orientation was carried out using the OrientationJ plugin of ImageJ to analyze birefringence patterns, as illustrated in Figure 5. The box plot represents the proportion of fibers within three orientation groupings with respect to the tendon long axes: ± 0°–10°, ± 10°–20°, and ± 20°–30° (measurements taken from n = 4 (E14), n = 3 (E16), n = 5 (E18), and n = 4 (E20)). **(D)** Representative images of sections across the developmental profile showing nuclei (DAPI), cytoplasm (GAPDH), and images merged (GAPDH/DAPI). Outline drawings of nuclei extracted from these images are shown in the bottom row. **(E)** Box plot representing cell density per 137.4* *μm^*2*^ regions quantified from multiple sections through the central body metatarsal tendons across development (n = 4 (E14), n = 3 (E16), n = 4 (E18), and n = 3 (E20) embryos). **(F)** Nuclear circularity increases (aspect ratio closer to 1) during tendon maturation (n = 219 (E14), n = 174 (E16), n = 190 (E18), and n = 151 (E20) cell measurements across replicates per stage). **(G)** Nuclear shape becomes more uniformly round during tendon maturation. Histogram representing the proportion of nuclear shapes (schematic representations with aspect ratio values shown below for each category) found in embryonic tendons. **(H)** The distance between cell nuclei in adjacent aligned columns increases significantly during development (n = 110 (E14), n = 96 (E16), n = 106 (E18), and n = 87 (E20) measurements across replicates/stage). Plot whiskers represent the minimum and maximum data values in all box plots. *p ≤ 0.05, **p ≤ 0.01, and ***p ≤ 0.001. Scale bar in **(A)** 200 *μ*m, **(B)** 100 *μ*m, and **(D)** 1 *μ*m.

From the earliest stage examined (E14), abundant collagen deposition was detected on longitudinal sections through metatarsal tendons (Figure 2A). To look closer at deposited collagen over time, picrosirius red staining (PSR) combined with polarized light microscopy (PLM) was used to analyze the spatial organization of the collagen network (Liu et al., 2021), allowing for quantitative analysis of collagen fiber alignment and reflecting the maturity of the deposited collagen (Patel et al., 2018); the color of the birefringent light has been previously taken to indicate increasing fiber thickness and maturity from green to yellow, orange, and red. Figure 2B shows representative images of PSR–PLM of metatarsal tendons across stages of development, indicating that collagen fibers shifted from a combination of green and yellow birefringence at E14 and E16 to predominantly yellow at E18 and E20. Some red birefringence is visible at the edges of tendons from E18 (Figure 2B). The analysis of the alignment of fibers shows progressively more alignment across late development (Figure 2C). Figure 2C represents the proportion of fibers in orientation groups ±10°, ±10°–20°, and ±20°–30° of the longitudinal axis across time and shows that the proportion of fibers in the ±10° category increases significantly (e.g., E14–E20: 41.7% ± 2.2% to 59.8% ± 2.1%; *p* < 0.001) with an accompanying reduction in the proportion of fibers in the third category, ±20°–30° (e.g., E14–E20: 13.3% ± 1% to 7.7% ± 0.6%; *p* < 0.001). This shift is also demonstrated by the proportion of fibers represented within ±30° of the longitudinal axis, increasing from 80% at E14, 83% at E16, and 88% at E18% to 91.5% at E20.

The number of cells per unit area within the tendons reduced from E14 to E18 (*p* = 0.044), E14 to E20 (*p* = 0.005), and E16 to E20 (*p* = 0.024) (Figure 2E), with the outline shape of the nuclei becoming more circular (value closer to 1) across development from E14 to E20 (Figure 2F), as illustrated by DAPI staining and nuclear aspect ratio (Figure 2D). At E14, the greatest diversity of nuclear shapes was identified, with only 20.1% of nuclei with an aspect ratio of <2 (i.e., more circular; lightest blue, as shown in Figure 2G). As development proceeds, the diversity in the nuclear shape reduces, with 91.4% of nuclei in the most circular outline category at E20 (Figure 1G). In addition to having a more uniform nuclear shape, the cells aligned into more organized columns, with the distance between nuclei in separate longitudinal columns increasing significantly between each pair of consecutive stages examined (Figure 2H). The mean distance between nuclei increased from 0.1 ± 0.005 *μ*m at E14 to 0.38 ± 0.02 *μ*m at E20 (*p* ≤ 0.001, Figure 2H). In summary, our data show that in embryonic chick ankle tendons, collagen fibers become more aligned, cell density reduces, the distance between columns of cells increases, and nuclei become more uniformly circular as they mature across late development.

### 3.2 Both rigid and flaccid paralysis during tendon maturation led to a reduction in the size of multiple tendons, while rigid paralysis also reduced tendon spacing

**FIGURE 3:**
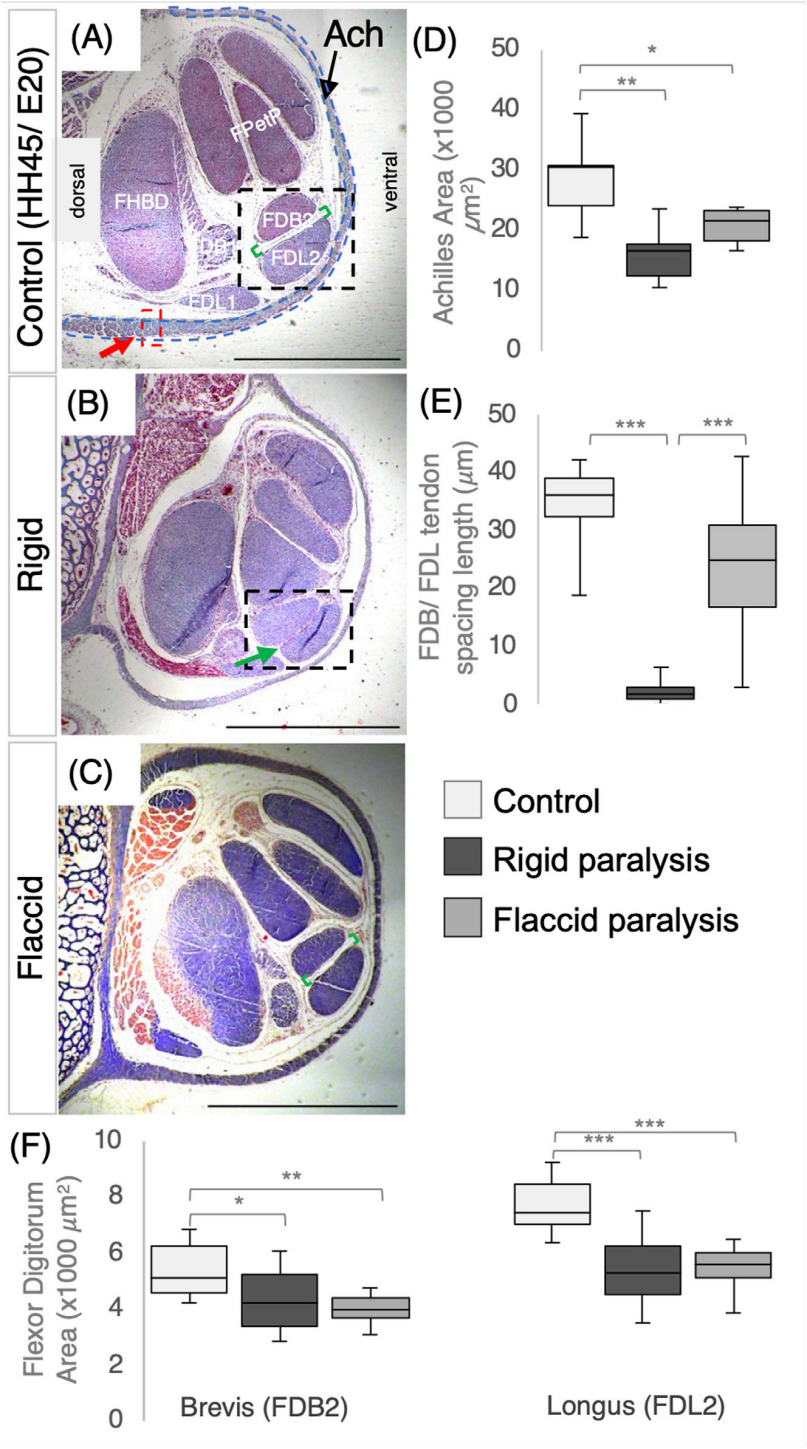
Immobilization by both rigid and flaccid paralysis alters tendon size, while only rigid paralysis alters spacing between adjacent tendons. **(A–C)** Representative cross sections of control **(A)**, rigid **(B)**, and flaccid-immobilized **(C)** tarsometatarsal regions of chick hindlimbs at embryonic day 20 (E20)/Hamburger and Hamilton stage 45 (HH45) stained with Masson’s trichrome. **(D)** Box plot of cross-sectional area of Achilles tendon, with area defined by the dashed blue line indicated in **(A)**. Both area and width were significantly reduced following immobilization (n = 7 (control), n = 6 (rigid), and n = 6 (flaccid)). **(E)** Box plot representing the inter tendon spacing (shown with green brackets in **(A–C)** across groups, a significant reduction in spacing following rigid immobilization is shown by the green arrow in **(B)** (n = 7 (control), n = 8 (rigid), and n = 7 (flaccid)). **(F)** Box plots of cross-sectional area measurements of FDB2 and FDL2, showing significant reductions following immobilization (n = 12 (control), n = 11 (rigid), and n = 8 (flaccid)). Plot whiskers represent the minimum and maximum data values in all box plots. *p ≤ 0.05, **p ≤ 0.01, and ***p ≤ 0.001. Scale bars **(A–C)** 1000 *μ*m. Achilles (Ach), flexor digitorum longus/brevis digit I (FDL/B1), flexor digitorum longus/brevis digit II (FDL/B2), flexor perforans et perforatus digit III and IV (FPetP), and flexor halluces brevis digits II, III, and IV (FHBD).

We previously showed that rigid paralysis induced by treatment with DMB from E14 to E17 caused a reduction in the size of metatarsal tendons (Peterson et al., 2021). In this section, we examine both rigid and flaccid (induced by treatment with PB) paralysis across an extended period during which the tendons are maturing (E16–E20) and also analyze their effects on multiple tendons. Both rigid and flaccid paralysis significantly reduced the size of the Achilles, flexor digitorum brevis, and flexor digitorum longus (FDL) digit II tendons at E20 (HH45) in the tarsometatarsal region (Figure 3). Significant decreases in the tendon size were observed through measurements of cross-sectional areas of the Achilles and both flexor digitorum tendons (Figures 3D, F). Effects on the Achilles tendon were also assessed through width measurements at defined points, confirming the finding of significant decreases in size following both types of paralysis (Supplementary Figure S1). We also examined the effect of paralysis at the knee joint region and observed a reduction in the width of the patellar tendon (ligament) (Supplementary Figure S1).

Paralysis also affected the spacing between the FDB and FDL tendons, but rigid and flaccid paralysis had different effects. Although there was a severe effect on spacing following rigid paralysis (Figure 3F; *p* < 0.0001; n = 8 immobilized embryos; n = 7 control; representative section shown in Figure 3B; green arrow), no significant reduction was observed following flaccid paralysis (n = 7 each of flaccid and control embryos). However, there was a larger variation in inter-tendon spacing measurements with flaccid paralysis (average of 23.9 ± 2.7 *μm* compared to 34.3 ± 0.7 *μm* in controls).

### 3.3 Rigid and flaccid paralysis have differential effects on collagen fiber alignment and cellular organization

**FIGURE 4:**
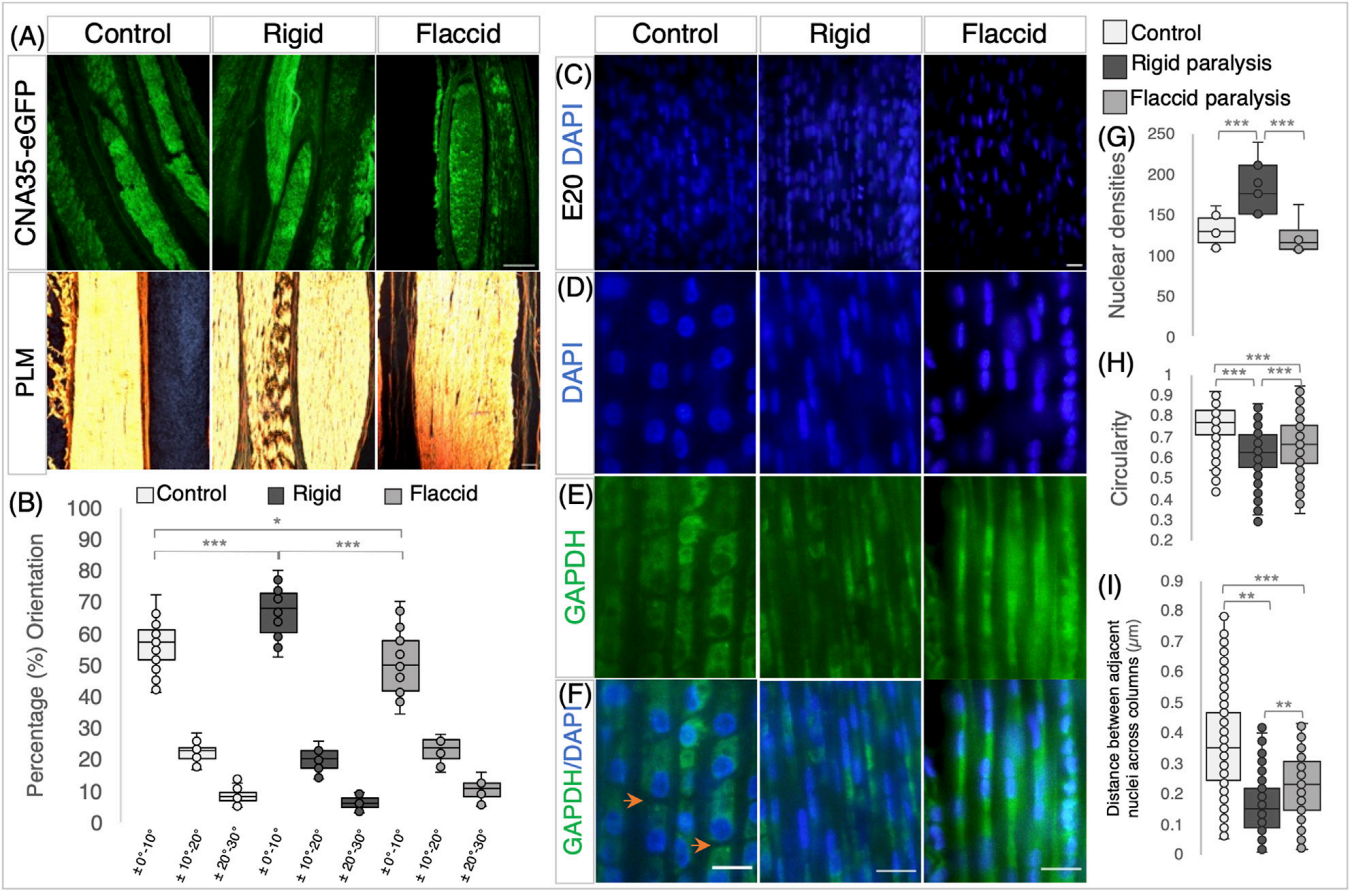
Rigid and flaccid paralysis have differential effects on collagen fiber alignment and cellular organization. **(A)** Collagen visualization using collagen-binding protein (CNA35-eGFP), with PLM, following picrosirius red staining (below) on longitudinal sections of metatarsal tendons at E20/HH45 and rigid and flaccid paralysis compared to control embryos, as indicated. **(B)** Quantification of collagen fiber orientation shows that fiber alignment is altered by immobilization, with increased alignment following rigid paralysis and less alignment with flaccid paralysis. The box plot represents the proportion of fibers within three orientation groupings with respect to the tendon long axes: ± 0°–10°, ± 10°–20°, and ± 20°–30° (measurements taken from n = 14 control, n = 7 rigid, and n = 5 flaccid immobilized embryos). **(C)** Representative views of DAPI-stained nuclei in E20 metatarsal tendons from control, rigid, and flaccid immobilizations within the 137.4* μm* ^*2*^ field of view used for cell counts. **(D–F)** Higher magnification views demonstrating nuclear shape. **(D)** GAPDH immunodetection (cytoplasm) **(E)** and merged images **(F)** across treatment groups. Orange arrowheads highlight separation between cells in the longitudinal axis **(F)**. **(G)** Cell density increased with rigid paralysis (n = 12 control, n = 7 rigid, and n = 6 flaccid immobilized embryos), whereas flaccid paralysis had no detectable effect. **(H)** Nuclear shape is less circular following immobilization compared to controls, with a more pronounced effect following rigid than flaccid paralysis (dots indicate cell replicates: n = 604 control nuclei from n = 12 embryos, n = 356 rigid nuclei from n = 7 embryos, and n = 308 flaccid nuclei from n = 6 embryos) (see D). **(I)** The distance between cell nuclei in adjacent aligned columns of cells within the tendon decreased significantly following immobilization with a greater reduction, following rigid paralysis (dots indicate individual measurements; n = 260 (control), n = 156 (rigid), and n = 106 (flaccid). Plot whiskers represent the minimum and maximum data values in all box plots. *p ≤ 0.05, **p ≤ 0.01, and ***p ≤ 0.001. Scale bar 200 *μ*m (top row), 100 *μ*m (bottom row) **(A)**, and 1 μm **(C–F)**.

Although there was no visual effect on deposited collagen following immobilization (Figure 4A), collagen fiber alignment within metatarsal tendons was differentially affected by rigid and flaccid paralysis from E16–E20. The intensity of fluorescent detection across conditions was not altered, and the birefringence pattern shows similar colors, with the majority of the fibers appearing yellow (with no discernible green), indicating intermediate maturity, and red (indicative of more mature) appearing at the margins of all tendons. Following fiber alignment analysis, the box plot shown in Figure 4B groups the proportion of fibers in orientation categories with respect to the long axis of the tendon. Rigid immobilization resulted in a higher proportion of fibers within ±10° of the long axis (66.9% ± 1.5% compared to 56.6% ± 1% in the control group; *p* < 0.001), while flaccid paralysis had the opposite effect with a lower proportion aligned (50.7% ± 2%, *p* = 0.011) (Figure 4B). The same patterns of change were observed in all categories.

When the effects of immobilization on tendon cellular organization were examined, both types of immobilization had effects, but to different extents, with rigid paralysis having the most pronounced effect. One distinct difference was observed in cell density analysis with rigid paralysis, leading to an increase in the number of nuclei per unit area compared to both normal moving controls (*p* = 0.007) and flaccidly (*p* = 0.027) immobilized tendons (Figures 4C, G). Flaccid paralysis had no significant effect. This could be indicative of a change in cell proliferation under rigid paralysis, but it must be seen in the light of the dramatic change in tendon size (e.g., a 51.1 ± 10.9% reduction in Achilles tendon cross-sectional area with a 130 ± 10% increase in cell density). Rigid and flaccid paralysis were both found to alter nuclear shape, with nuclei becoming more elongated compared to controls at E20/HH45 (*p* < 0.001 in both immobilization modes); however, although nuclei under flaccid conditions are elongated, they are less than rigid nuclei (Figures 4D, H, P = 0.001). Although nuclei are arranged in columnar parallel patterns across treatment groups, the shape of cells following immobilization was disturbed, as revealed by the cytoplasmic staining of GAPDH (Figure 4E). In control tendons, the tenocytes are regularly stacked and clearly defined with a cuboidal shape and clear separation between neighboring cells (Figure 4F; orange arrowheads), whereas following immobilization, there is no visual separation between cells within longitudinal columns. Additionally, the transverse distance between nuclei in adjacent aligned columns is reduced under both immobilization conditions compared to control (rigid, 0.16 ± 0.01 *μ*m and flaccid, 0.22 ± 0.01 *μ*m compared to control, 0.36 ± 0.01 *μ*m ((*p* ≤ 0.001, Figure 4I), with rigid paralyzed embryos being more severely affected.

In line with the observed loss of regular cellular organization, the diversity of nuclei shapes significantly increases following immobilization (Supplementary Figure S2). The disruption of collagen fiber alignment and cellular organization with immobilization strongly indicates that the structural organization of tendons is reliant on movement.

### 3.4 Components of YAP signaling pathway are expressed in the late-maturing tendon with alteration of target gene expression levels following immobilization

**FIGURE 5:**
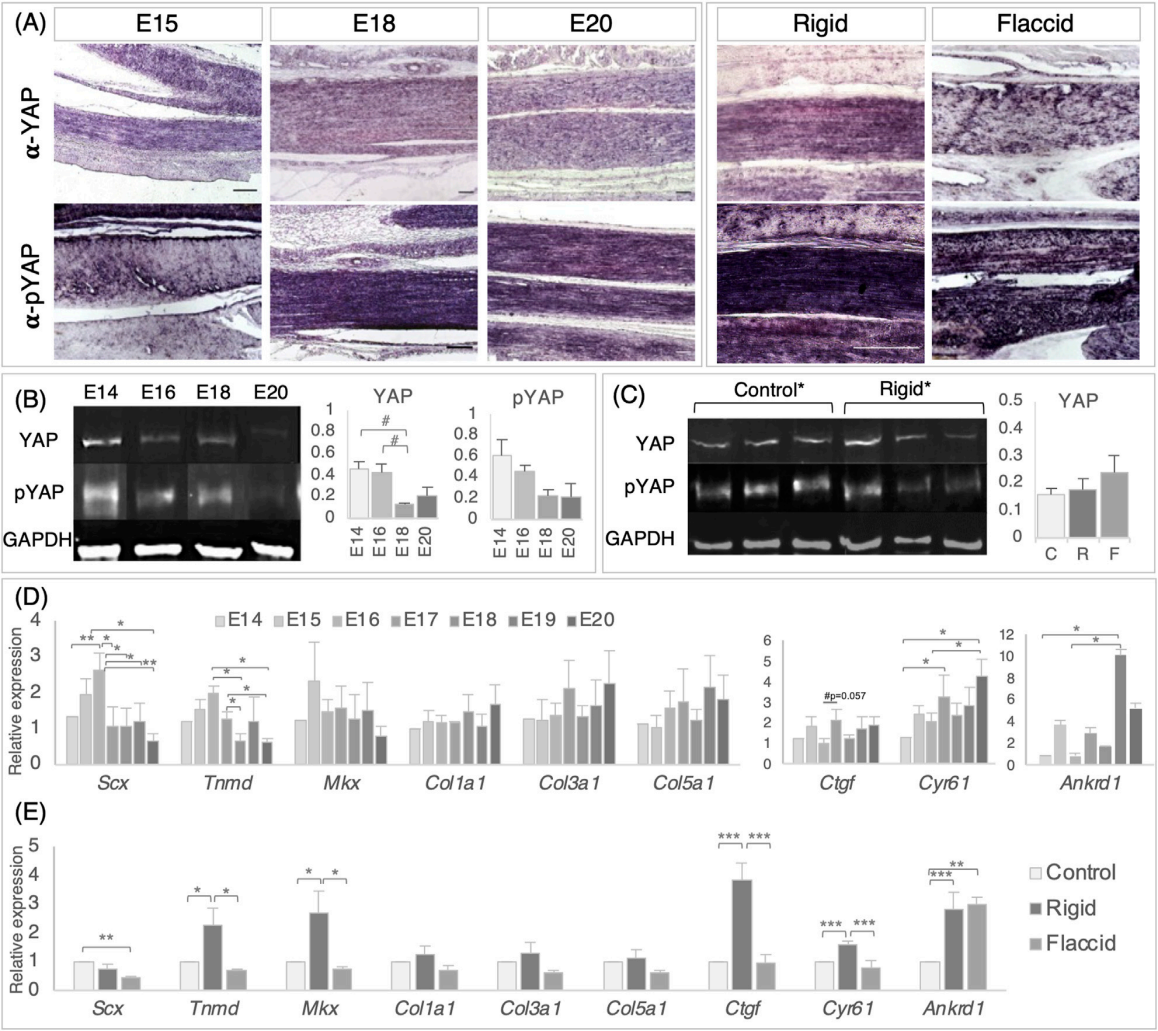
Detection and quantification of YAP signaling pathway components in the late stage tendon, across development and following immobilization, shows that target gene expression is altered following immobilization. **(A)** Immunodetection of YAP and pYAP proteins show widespread localization throughout late stage developing metatarsal tendons across developmental timepoints and following immobilization. Example longitudinal sections are shown at E15, E18, and E20 and following rigid and flaccid paralysis at E20 (E20 normal development and E20 control samples were separately analyzed and were equivalent throughout, so the E20 column represents both). **(B)** Protein quantification of YAP (65 kDa) and pYAP (65 kDA) by Western blot, normalized to GAPDH (36 kDa) levels, over developmental timepoints, reveals a trend of reduced mean YAP protein levels between E14–E18 (#*p* = 0.067) and E16–E18 (#*p* = 0.059), but with no significant difference. Example protein bands for each stage (E14, E16, E18, and E20) are shown. The histograms show YAP and pYAP absolute protein levels (n = 3 (E14, E18, and E20) n = 5 (E16)). **(C)** YAP and pYAP quantification following immobilization show no significant differences in protein levels following immobilization (three example replicate protein bands shown for control and rigid groups). The histogram shows data for YAP {n = 15 [control (C)], n = 11 [rigid (R)], and n = 3 [flaccid (F)]}. **(D)** Relative gene expression levels in metatarsal tendons across developmental timepoints, as indicated for *scleraxis*, *tenomodulin*, *Mohawk*, *collagen 1*, *collagen 3*, and *collagen 5*, together with YAP pathway target genes *Ctgf*, *Cyr61*, and *Ankrd1*. All data were normalized to *GAPDH* expression and presented relative to E14 expression levels (n = 3–6). Note that YAP target genes *Cyr61* and *Ankrd1* showed significantly increased expression in later stages of development (n = 3–6). **(E)** Relative gene expression following rigid (n = 11–13) and flaccid immobilization (n = 6). All data were normalized to *GAPDH* levels and expressed relative to control (n = 20). Although the expression levels of collagen genes were not altered by immobilization, both tendon regulatory genes (Tnmd and Mkx) and YAP target genes were upregulated by rigid immobilization but not by flaccid immobilization. Plot whiskers represent the minimum and maximum data values in all box plots. ^#^ *p ≤ 0.1*, *p ≤ 0.05, **p ≤ 0.01, and ***p ≤ 0.001. Scale bars 100 *μ*m.

To investigate the YAP signaling pathway as a possible mechanoregulator of tendon maturation, we set out to establish whether YAP pathway components are localized in the tendon during late development and whether levels are altered following immobilization, comparing rigid and flaccid paralysis. Using immunodetection on tissue sections, we could readily detect both the active form (YAP) and inactive form (pYAP) in the metatarsal tendons across timepoints following immobilization (Figure 5A; n = 8 rigid and n = 6 flaccid biological replicates histologically analyzed). No consistent differences in localization patterns were observed; any apparent differences between adjacent tendons are likely due to the precise plane of the section and were not consistently reproduced (n = 12). This was supported by the quantification of YAP and pYAP absolute protein-level accumulation via Western blot, which showed no significant change in levels of either protein, but it revealed a trend of reduction in mean YAP levels across the later stages of development between E14–E18 and E16–E18 (Figure 5B, #*p* = 0.067 and *p* = 0.059, respectively). To check for a possible shift in the relative levels of YAP and pYAP, the ratios of active YAP normalized by total YAP (YAP + pYAP) levels were examined and showed no change over time (YAP/(YAP + pYAP) ratios: E14, 0.44 ± 0.06; E16, 0.52 ± 0.007; E18, 0.38 ± 0.05; and E20, 0.53 ± 0.16). Similarly, no quantitative differences were detected for YAP or pYAP protein levels following immobilization (Figure 5C, histogram representative of n = 15 controls, n = 11 rigid n = 3 flaccid embryos screened for YAP; YAP/(YAP + pYAP) = 0.51 ± 0.04 for control and 0.48 ± 0.04 for immobilized).

Gene expression profiling of *in vivo* tarsometatarsal chick tendons every 24 h from E14 to E20 show that the relative expression of *Scx* increases between E14 and E16 (*p* = 0.007) and then reduces from E16 to E17–E20 inclusively (Figure 5D, (E16–E17, *p* = 0.01; E16–E18, *p* = 0.01; E16–E19, *p* = 0.026; and E16–E20, *p* = 0.001)). Similar decreases in *Tnmd* expression were observed between E16 to E18 and E20 (*p* = 0.02 and *p* = 0.01, respectively) and E17 to E18 and E20 (*p* = 0.02 and *p* = 0.01, respectively, Figure 5D). No significant changes in gene expression were observed for *Mkx* or tendon-specific collagen genes (*col1a1*, *col3a1*, or *col5a1*) across the late stage of tendon development. Interestingly, YAP target genes were found to be expressed across developmental time, from E14 to E20, indicating pathway activity, with *Cyr61* and *Ankrd1* more highly expressed at later stages (Figure 5D). An increase in *Cyr61* was observed between E14–E17 (*p* = 0.044), E14–E20 (*p* = 0.002), and E16–E20 (*p* = 0.022), and increases in *Ankrd1* expression were observed from E14–E19 and E16–E19 (*p* = 0.042 and *p* = 0.03, respectively) (Figure 5D). The detection of pathway components and quantitative changes in expression levels indicate that the pathway may be modulated in this developing system.

Multiple disturbances in gene expression levels were detected following immobilization (Figure 5E). Rigid immobilization resulted in more gene disturbances than flaccid immobilization when compared to control expression (5 and 2 genes, respectively), with four of the rigid disturbances identified also differing from flaccid expression (*Tnmd*, *Mkx*, *Ctgf*, and *Cyr61*), suggesting that rigid and flaccid immobilization may affect the YAP pathway differently (Figure 5E). Alterations in the expression of tendon-specific genes *Scx*, *Tnmd*, and *Mkx* were observed; a 2.21-fold decrease was observed in the expression of *Scx* with flaccid paralysis (*p* = 0.006), whereas 2.29-fold and 2.68-fold increases in the expression of *Tnmd* and *Mkx* were identified with rigid paralysis (*p* = 0.01 and *p* = 0.012, respectively, Figure 5E). No expression changes were observed in any of the collagen genes screened. Rigid immobilization led to the increased expression of YAP target genes *Ctgf* (*p* = 0.000002), *Cyr61/Ccn1* (*p* = 0.000117), and *Ankrd1* (*p* = 0.000263). Flaccid immobilization led to an increased expression of *Ankrd1* only (*p* = 0.001), while both *Ctgf* and *Cyr61* expression levels were not different from control levels but significantly less than following rigid immobilization (Figure 5E). The observed increased activity of established pathway target genes in rigidly immobilized tendons suggests that the YAP pathway output is tightly controlled during normal development, mediated by mechanical cues and/or biophysical changes, and impacted by immobilization; when constant static muscle forces are imparted, but dynamic stimulation is lost (rigid paralysis), the effect is strongest, compared to flaccid paralysis, which removes both dynamic and static muscle forces.

## 4 Discussion

To understand better the tissue level and cellular and molecular changes that take place during tendon maturation, we report a two-pronged experimental approach: 1) we profiled changes *in vivo* across the relevant period of development, E14–E20 in the chick, and 2) we immobilized embryos, comparing rigid and flaccid paralysis, to excavate more precisely how maturation is dependent on embryo movement. We show increasingly mature and aligned collagen fibers across key developmental timepoints. Furthermore, cell density decreases, and cell shape becomes increasingly cuboidal, with cells stacked end-to-end into parallel columns within the developing tendon, resulting in increased distance between columns. We show that both rigid and flaccid paralysis affect tendon maturation, but their effects are distinct; while both affect tendon size, only rigid paralysis significantly affects flexor tendon spacing, increasing fiber alignment and cell density. Rigid paralysis has a more extreme effect on the nuclear shape and spacing between tenocyte columns. To explore the mechanisms involved in the cellular response to mechanical stimulation from movement during tendon maturation, we examined the components of YAP signaling and observed that both YAP (active) and pYAP (inactive) are readily detected in the late developing tendon, although there were no quantitative differences in the levels across developmental timepoints or under immobilization. However, YAP target gene expression was upregulated across late development, indicating that the pathway is modulated. Furthermore, following rigid paralysis, expression levels of YAP target genes were further elevated implicating YAP signaling in the mechanoresponse during late tendon development for the first time.

Immobilization of chick embryos across late stages of tendon development (from E15 or E17) previously showed that the mechanical properties of the tendon are affected, with a reduction in the modulus following rigid (Pan et al., 2018; Peterson et al., 2021; Peterson et al., 2024) and flaccid paralysis (Peterson et al., 2021; Peterson et al., 2024). Indeed, we previously showed that flaccid paralysis led to a stronger reduction in modulus than rigid paralysis (Peterson et al., 2021). In this study, we show further distinction between the effects in several respects, with, most notably, rigid paralysis alone causing an increase in fiber alignment and cell densities and a greater effect on YAP target gene expression. In this experimental system, rigid paralysis is induced by treatment with DMB, a neuromuscular blocking agent that induces tetanus (Osborne et al., 2002), with sustained contraction of all skeletal muscles, i.e., dynamic forces are removed, but static forces remain constant (Nowlan et al., 2008), albeit with reduced force due to reduced muscle size and contractile properties (Reiser et al., 1988). In the case of flaccid paralysis induced by PB treatment, both dynamic and static forces are absent (Osborne et al., 2002; Rolfe et al., 2017). Therefore, unique or exaggerated effects resulting from rigid paralysis are likely due to the constant strain applied and/or the lack of dynamic stimulation, whereas stronger effects of flaccid paralysis indicate features that are more sensitive to the absolute reduction in force experienced. For example, the exaggerated alignment of collagen fibrils and the increase in cell density respond to inappropriate constant mechanical force from muscles in tetanus, whereas the reduction in fiber alignment and increased effects on reduction in tendon modulus by flaccid paralysis (Peterson et al., 2021) indicate a reliance on the level of force applied. This suggests that tendon maturation depends not only on the level of muscle force applied but also requires dynamic stimulation for specific aspects. A milder effect of flaccid paralysis compared to rigid paralysis has also been reported previously in the developing chick spine (Rolfe et al., 2017) and joint formation (Osborne et al., 2002). However, the difference between flaccid and rigid paralysis may be dependent on timing, as both forms of immobilization of chicken embryos at a slightly earlier timepoint (E13) produced similar deficits in tendon tissue mechanics (Peterson et al., 2024).

Collagen fibers run parallel to the tissue’s long axis in tendons and ligaments, a key factor contributing to their high tensile strength and ability to withstand large forces (Birk et al., 1995; Birk et al., 1991). Therefore, the structural organization of collagen is an important aspect of the system to monitor. Birk et al. (1995) showed a large step increase in the length of collagen fibrils by lateral fusion at E17 in the chick (Birk et al., 1995) coincident with increased tensile strength (McBride et al., 1988), while our previous study showed an increase in the fibril:tissue strain ratio, consistent with an increase in the length of the collagen fibrils (Peterson et al., 2021; Peterson et al., 2024). Here, visualization of collagen deposition using a fluorescently tagged collagen-binding protein showed readily detectable collagen across the developmental profile. Using PLM to assess fiber orientation showed an increasing proportion of fibers within ±10° of the tendon long axis across time, increasing from approximately 40%–60% between E14 and E20 (Figure 2C). Rigid and flaccid immobilization both affected orientation but in opposite directions; rigid immobilization increased the proportion aligned, while flaccid immobilization led to a decrease (Figure 4B). This shows that muscle contraction is required for the optimum alignment of fibers, with the constant static force of rigid paralysis causing increased alignment. This key aspect of collagen structural change, so important for the mechanical properties of the tissue, is stimulated by normal embryo movement.

The collagen fibrils are deposited by tenocytes, but we know very little about how this later aspect of tenocyte differentiation is regulated or even how this important maturation process is achieved by the cells; therefore, we aim to examine cellular organization in the maturing tendon in particular. McBride et al. (1985) showed preliminary data (n = 2 per stage) indicating that the cell volume fraction decreases across maturation timepoints in the chick tendon (McBride et al., 1985), and Kalson et al. (2015) showed cell per unit volume reduction across late embryonic stages and up to 6 weeks post-natal in the mouse tail tendon (Kalson et al., 2015). We verified that the number of cells per area in chick metatarsal flexor tendons decreased between E14 and E18 and E20 (Figure 2E) and that rigid paralysis led to an increase in the cell density at E20, reaching levels observed at E16 in normal development when immobilization treatments began (Figure 4G). Although only rigid paralysis affected cell density, it shows that it is responsive to the mechanical environment. Visualizing cells within the developing tendon showed a number of striking changes in spatial organization across developmental time. Although cells were already aligned longitudinally at E14, the linear arrangements were narrow and close together, with no clear distinction between the cytoplasm of linearly adjacent cells, apparently elongated and overlapping. The nuclei were extremely elongated, with long axes running in the direction of the linear cellular arrangements. The nuclei became rounded as development progressed to E20, the linear columns further separated, and there were clear interfaces between the cytoplasm of linearly adjacent cells (Figures 2D, 3E). This indicates that cells are organized into discrete cuboidal, stacked arrangements by E20. This is reminiscent of observations from serial block-face scanning EM in mouse-tail tendons across a later time frame of up to 6 weeks post-natal (Kalson et al., 2015). Kalson et al. (2015) proposed that the arrangement of cells stacked end-to-end is important for neighboring cells to define sections of continuous vertical channels for the assembly and growth of collagen fibrils; i.e., this arrangement is important for controlling collagen maturation (Kalson et al., 2015). We show a similar arrangement in in chick tendons but achieved at an earlier developmental time point. The increased distance between the columns of cells up to E20 coincides with the increased diameter of fibers (Kalson et al., 2015; Peterson et al., 2021), which is interpreted as creating space for more ECM deposition and accounting for the reduction in the cell volume-to-ECM ratio. Importantly, we found that both flaccid and rigid immobilization caused retardation in this progression toward a more mature arrangement, with rigid immobilization having a stronger effect than flaccid immobilization (Figure 4I). Therefore, one of the impacts of the dynamic mechanical environment generated by the movement is the correct spatial organization of tenocytes, which is necessary for the maturation of the ECM.

A central objective of this study was to investigate YAP signaling as a candidate biological mechanism that integrates mechanical signals during the critical period of tendon maturation. It is a prime candidate mechanotransduction pathway to investigate due to its proven role in numerous biological contexts (Deng et al., 2016; Dupont et al., 2011; Shea et al., 2020; Wada et al., 2011), with emerging evidence of a potential role in tenogenesis *in vitro* (Li et al., 2021; Lu et al., 2023; Tao et al., 2021; Tomás et al., 2019; Xu et al., 2022). In this study, we showed the localization of YAP and pYAP in the tendon across stages, and the expression of YAP target genes revealed significant increases across the period of maturation, indicating that the pathway is active and modulated (Figure 5). Following rigid immobilization, there was an increase in the levels of all three target genes assayed (*Ctgf*, *Cyr61*, *and Ankrd1*) together with increases in tendon regulatory genes *Tnmd* and *Mkx*, with no increase in collagen genes and a decrease in *Scx*. Flaccid paralysis only led to a significant increase in Ankrd1 expression, indicating that the activity of the pathway was more highly impacted by rigid paralysis (Figure 5E). Immobilization from an earlier developmental timepoint might lead to greater disturbance, but this study was designed to focus on the biomechanical transition in tendon properties in later development. A quantitative investigation of nuclear versus cytoplasmic YAP localization was not feasible in embryonic tendon tissue due to a number of architectural features of the tissue. While YAP-positive nuclei are evident under all conditions, the cytoplasm is also widely stained without a clear definition between individual cells. Analysis was also hampered by the high density of cells in parallel alignment and the shape of the cells, especially in immobilized tissue (Figure 4E). This is the first study to investigate YAP signaling in the embryonic tendon with movement manipulation. Our data suggest that the YAP pathway output is tightly controlled during normal development and is impacted by mechanical cues, most strongly in the constant force environment of rigid paralysis. It is somewhat surprising that there is an increase in target gene expression across development, and yet paralysis leads to a further increase in expression levels rather than a decrease. This may be due to the constant static stimulation of the rigid state. The increase in *Ankrd 1* expression in flaccid paralysis may be due to different regulator inputs for this particular gene. Indeed, each of these recognized YAP target genes is known to be influenced by other pathways, which may also be modulated by the mechanical environment and contribute to integrated differential regulation, particularly Wnt, TGFβ, and notch signaling (Konsavage and Yochum, 2013; Tao et al., 2014; Tschaharganeh et al., 2013; Varelas et al., 2010).

A surprising finding in this study is the change in the shape of tenocyte nuclei as development proceeds from E14 to E20, going from extremely elongated to more round (Figure 2F). It has been extensively reported that nuclei in adult tendons are elongated (Freedman et al., 2022); however, tendons with more elongated nuclei are seen at 8 and 19 months compared to 1-month-old rat Achilles tendons (Freedman et al., 2022). Our finding is in contrast to that of the embryonic chick jaw-closing tendon, TmAM, which shows nuclear elongation as development proceeds (Korntner et al., 2022). This difference could be due to the distinct anatomical location of these tendons, and perhaps an accelerated differentiation progression is required for jaw tendon function at birth for feeding. It is possible that the nuclear shape reflects the mechanoregulator environment; the nucleus is the stiffest organelle and is known to respond to mechanical forces (reviewed by Dahl et al., 2008). Signaling from the ECM through integrins and focal adhesion kinase can lead to an alteration in the shape of the nucleus. Interestingly, it has been shown that altering the shape of the nucleus by plating cells on a micro-patterned substrate affected collagen 1 synthesis (Thomas et al., 2002). These observations will be further explored by comparing nuclear shapes in developing mouse tendons.

This study has provided insight into the structural, cellular, and molecular changes in embryonic chick tendons during late embryonic development and has expanded understanding of the impact of immobilization on tendon maturation. We have shown for the first time that YAP signaling is altered following immobilization across the important period of the rapid maturation of tendon mechanical properties. Future work will focus on the manipulation of the signaling pathway to further test its specific role in tendon maturation. We also describe several aspects of cell organization and collagen deposition within the tendon that change across this period and are sensitive to movement. Collagen fiber alignment and the organization of cells into end-to-end stacked columns, with increasing distance between adjacent columns over time, were also shown to be altered following immobilization with different effects of rigid and flaccid paralysis. This organization resembles cellular arrangements observed at later stages in the mouse tendon, which are postulated to be important in fibril growth. We conclude that tendon maturation requires force exerted by associated muscles, with specific aspects requiring a highly controlled level of dynamic stimulation. This study employs a developmental approach to understanding how tendons are constructed and will form an important basis for future work to engineer improved tensile load-bearing tissues. It illuminates the mechanistic basis of mechanoregulation during development as an important step in knowing what molecular cues and signals to provide during tissue construction *in vitro.*

## Data Availability

Data are presented in the paper and Supplementary Material: further enquiries can be directed to the corresponding author.
